# Stress‐induced cardiovascular morbidity: Past, present and future

**DOI:** 10.1113/EP091468

**Published:** 2023-09-15

**Authors:** Aoife D. Slyne, Fiona B. McDonald, Ken D. O'Halloran

**Affiliations:** ^1^ Department of Physiology, School of Medicine, College of Medicine & Health University College Cork Cork Ireland

**Keywords:** blood pressure, gestational stress, paraventricular nucleus

1

Normal fetal development is critical to life‐long health. Stress experienced in utero can have lasting effects, increasing the risk of developing various pathologies in later life, including cardiometabolic diseases. Gestational stress disrupts neural programming. Obstructive sleep apnoea is commonly experienced in pregnant women, resulting in exposure to intermittent hypoxia, both for the mother and for the fetus. Previous pre‐clinical studies have demonstrated that gestational intermittent hypoxia (GIH) leads to a sex‐specific increase in blood pressure, with adverse outcomes in adult male offspring (Song et al., 2022).

In this issue of *Experimental Physiology*, Ambrozio‐Marques et al. (2023) examined the proclivity for GIH to activate key stress sites in the brain, with a focus on the amygdala and paraventricular nucleus of the hypothalamus (PVN). Pregnant rats were exposed to an established protocol of intermittent hypoxia for the latter half of pregnancy. Subsequently, in adult offspring, FosB immunolabelling was used as a marker of neuronal activation, to examine if exposure to GIH primes neural circuitry pivotal to integrated stress responses. Outcomes were compared in adult male and female rats. The authors determined that GIH augments basal activation of the PVN of adult male, but not adult female offspring. No differences were observed in the amygdala. The results reveal a persistent, sex‐specific effect of GIH on the neuroendocrine axis, offering a plausible mechanism by which GIH disturbs behaviour and blood pressure in adulthood.

The PVN is a critical node of the stress axis and a major integrative site of cardiovascular control, subserving autonomic and neuroendocrine functions, and controlling blood pressure. Other models of early life stress, including gestational stress, have demonstrated persistent hypothalamic–pituitary–adrenal activation with increased levels of circulating stress hormones and cardiorespiratory dysfunction, including the manifestation of a hypertensive phenotype. It will be important to fully establish if GIH follows a similar trajectory, which has major implications for our consideration of the risk to the adult of antecedent stress experienced by the fetus arising from maternal obstructive sleep apnoea.

Exposure to intermittent hypoxia during pregnancy is potentially stressful to the dam, such that the substrate for potentiated stress responses in male GIH offspring could relate to direct GIH‐related stress on the fetus and/or indirect mechanisms secondary to direct effects of hypoxic stress in the pregnant dam. The persistent nature of PVN activation suggests that in addition to GIH‐induced aberrant plasticity in the neuroendocrine circuitry, there may also be epigenetic changes caused by exposure to intermittent hypoxia in utero. Although the inheritance pattern is unknown, the risk of hypertension in offspring is approximately two times higher when one parent has hypertension (Jang et al., 2023). Therefore, GIH has deleterious consequences for the fetus as an adult but may also transfer trans‐generationally to future offspring.

Beyond the risk for high blood pressure, a growing body of literature reveals that fetal hypoxia programmes mitochondrial pathways contributing to a range of cardiac abnormalities in adult male offspring. Gestational hypoxic stress affects mitochondrial reactive oxygen species generation and cardiac mitochondrial respiration in fetal rat hearts in a sex‐dependent manner (Smith et al., 2022), with lasting divergent responses between sexes in mice at 8 months of age (Hellgren et al., 2021). Specifically, in adult male mice, gestational hypoxic stress resulted in decreased protein abundance of electron transport complexes, impaired oxidative phosphorylation and increased production of hydrogen peroxide, whereas remarkably in adult female mice from hypoxic pregnancies, complex IV activity was increased with a resultant increase in mitochondrial respiration and decreased levels of hydrogen peroxide (Hellgren et al., 2021). Gestational hypoxia also causes pulmonary and systemic vascular dysfunction, including cerebrovascular dysfunction, revealing widespread ravaging effects of early life stress on integral components of the cardiovascular system.

The full extent of cardiometabolic consequences of GIH must be established, with a delineation of mechanisms underpinning lasting aberrant responses, so that we have a complete portfolio of the pathophysiological consequences of gestational hypoxic stress. Moreover, the fundamental basis for sexual dimorphism in long‐term outcomes, revealing a striking male vulnerability, must be established. There is widespread recognition of the cardiovascular and metabolic risks associated with sleep‐disordered breathing. Now those concerns extend to pregnant women, their male children and perhaps grandchildren. Emerging findings suggest that greater recognition and treatment of maternal sleep‐disordered breathing may decrease some of the global burden of cardiovascular disease.

## AUTHOR CONTRIBUTIONS

All authors contributed to the drafting of the manuscript. All authors approved the final version of the manuscript.

## CONFLICT OF INTEREST

The authors declare no conflicts of interest.
